# Advances in CAR-NK cell therapy for lung cancer: is it a better choice in the future?

**DOI:** 10.3389/fonc.2024.1390006

**Published:** 2024-05-28

**Authors:** Fengqin Liu, Xia Miao, Lu Han, Xiao Song

**Affiliations:** ^1^ The Third Department of Geriatrics, Weifang People’s Hospital, Weifang, Shandong, China; ^2^ Central Supply Service Department (CSSD), Weifang People’s Hospital, Weifang, Shandong, China; ^3^ Department of Gastroenterology, Weifang People’s Hospital, Weifang, Shandong, China

**Keywords:** lung cancer, CAR-NK, immunotherapy, therapy, target

## Abstract

Lung cancer remains one of the leading causes of cancer-related mortality worldwide necessitating the development of innovative therapeutic strategies. Chimeric antigen receptor (CAR) natural killer (NK) cell therapy represents a promising advancement in the field of oncology offering a novel approach to target and eliminate tumor cells with high specificity and reduced risk of immune-related adverse effects. This paper reviews the mechanism, potential targets, and recent advances in CAR-NK cell therapy for lung cancer, including the design and engineering of CAR-NK cells, preclinical studies, and the outcomes of early-phase clinical trials. We highlight the unique advantages of using NK cells, such as their innate ability to recognize and kill cancer cells and their reduced potential for inducing graft-versus-host disease (GvHD) and cytokine release syndrome (CRS) compared to CAR T-cell therapies. Results from recent studies demonstrate significant antitumor activity in lung cancer models with improved targeting and persistence of CAR-NK cells observed *in vitro* and *in vivo*. Finally, we discuss the challenges in optimizing CAR-NK cell therapies, including the potential resistance mechanisms. The paper concludes with an outlook on the future directions of CAR-NK cell research and its implications for lung cancer treatment emphasizing the importance of continued innovation and collaboration in the field.

## Introduction

Lung cancer remains a major global health challenge accounting for a significant portion of cancer deaths worldwide. As of 2023, there occurred approximately 238,340 new cases of lung cancer (117,550 in men and 120,790 in women), and it was estimated to cause 127,070 deaths from lung cancer (67,160 in men and 59,910 in women) (1). From a global perspective, as of 2020, it was estimated to cause about 1.8 million deaths representing 18% of all cancer deaths (2). Lung cancer is the second most common cancer in both men and women in the U.S. after prostate and breast cancer, respectively (1). At diagnosis, lung cancer is classified based on the type of cells the tumor is derived from. There are two major types of lung cancer categorized by the pathological description of the malignant cells as follows: small-cell lung cancer (SCLC; 15% of cases) and non-small-cell lung cancer (NSCLC; 85% of cases) (3). The primary risk factor for lung cancer is smoking tobacco, which is responsible for approximately 85% of all cases. This includes cigarettes, cigars, and pipes. Other risk factors include exposure to secondhand smoke, occupational hazards (such as asbestos, radon, and certain chemicals), air pollution, hereditary cancer syndromes, and previous chronic lung diseases (3).

Physical examinations, imaging (chest X-rays, CT scans, MRI), bronchoscopy, biopsies, and molecular testing are often used to diagnose lung cancer and determine the specific subtype (NSCLC vs. SCLC). In addition to the classification of lung cancer, the staging of lung cancer is also particularly important for the selection of treatment options. Common treatments include surgery, radiation therapy, chemotherapy, targeted drug therapy, and immunotherapy (3). Common immunotherapies include adoptive cell therapy, checkpoint blockade immunotherapies (like drugs targeting the PD-1/PD-L1 pathway), and cancer vaccines (4). Adoptive cell therapies are quite popular in the field of lung cancer treatment, including chimeric antigen receptor T-cell (CAR T) therapy, T-cell receptor (TCR) therapy, and tumor-infiltrating lymphocyte (TIL) therapy (5). These approaches involve using a patient’s own immune cells, modified and expanded in the laboratory, to target and destroy cancer cells. However, due to the limitations of T cells themselves, such as limited sources and easy triggering of inflammatory factor storms, researchers have turned their attention to other immune cells that can be modified, such as NK (natural killer) cells (6). As a new star in cell therapy, CAR-NK therapy has attracted much attention in recent years. This study intends to discuss the possibility, advantages, and challenges of using CAR-NK in the treatment of lung cancer from multiple dimensions.

## Mechanism of CAR-NK cell therapy

**Figure 1:**
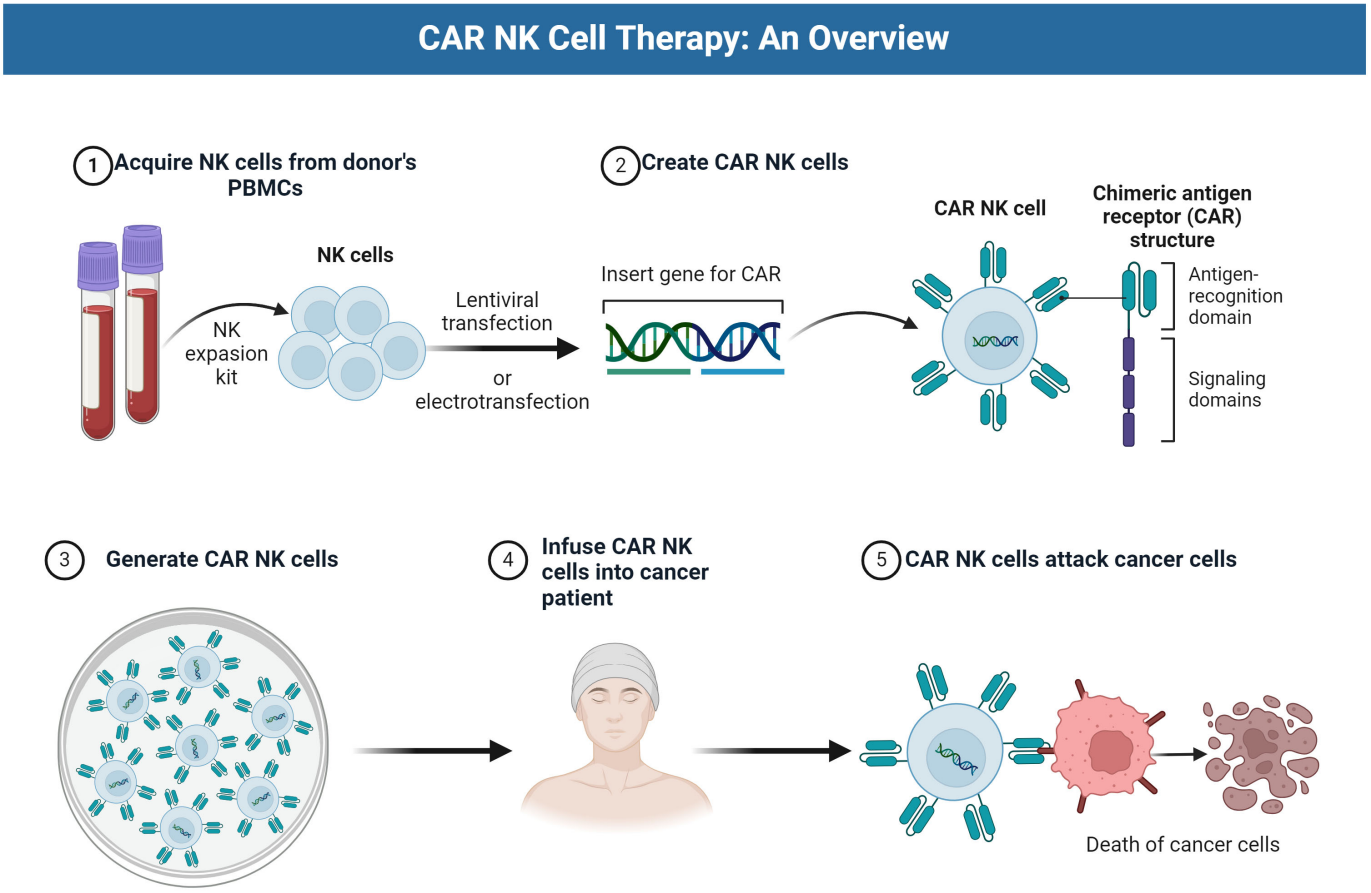
The CAR NK cell therapy process.

NK cells are a type of lymphocyte in the immune system that play a crucial role in the body’s defense against tumors and viral infections. NK cells are functionally similar to CD8+ cytotoxic T cells and kill target cells through similar cytotoxic mechanisms, but lack a somatically rearranged and antigen-specific TCR. Tumor cells with lower expression of human leukocyte antigen (HLA) may be more susceptible to NK cell killing due to a reduced KIR-mediated inhibition (7). NK cells used in therapy can be derived from various sources like peripheral blood mononuclear cells, cord blood, immortalized cell lines, hematopoietic stem and progenitor cells (HSPCs), and induced pluripotent stem cells (iPSCs) (6, 7). Allogeneic NK cells are often preferred over autologous cells due to the dysfunctional phenotype of autologous NK cells in cancer patients. NK cell lines like NK-92MI have also been approved to load the CAR structure to create a CAR-NK therapeutic system and apply it clinically. This further increases the source and availability of NK cells (8). NK-92 cells, in contrast to NK cells from other sources, have predictable expansion kinetics and can be grown in bioreactors that produce billions of cells within a couple of weeks. In addition, NK cell lines can easily be transduced by physical methods with high efficiency. CAR-expressing NK-92 has been generated to target several cancer surface receptors, such as CD19 (a type of B-cell receptor) (9), human epidermal growth factor receptor 2 (HER2/ErbB2) (10), and epidermal growth factor receptor (EGFR, aka HER1) (11), and many of these engineered NK-92 cells are currently in clinical trials for the treatment of cancer. CAR-NK cells are engineered to recognize and attack specific antigens present in cancer cells. **Figure 1** shows the CAR-NK cell therapy process using PBMC as an example.

## Potential CAR NK lung cancer targets and preclinical experimental results

Immunotherapy for lung cancer is very popular and has achieved great success. Population-level mortality from NSCLC in the United States fell sharply from 2013 to 2016, and survival after diagnosis improved substantially (12). Several different genetic mutations may arise in lung tumors, and some may be more likely to cause the cancer cells to spread to other parts of the body. As an example, mutations of epidermal growth factor receptor (EGFR) have been identified as key drivers of metastasis, and safe, efficacious therapies have been developed to target EGFR mutations and inhibit cancer spread (13). These targets can also be used as a basis to design the antigen-recognition region of CAR-NK therapy.

**Figure 2:**
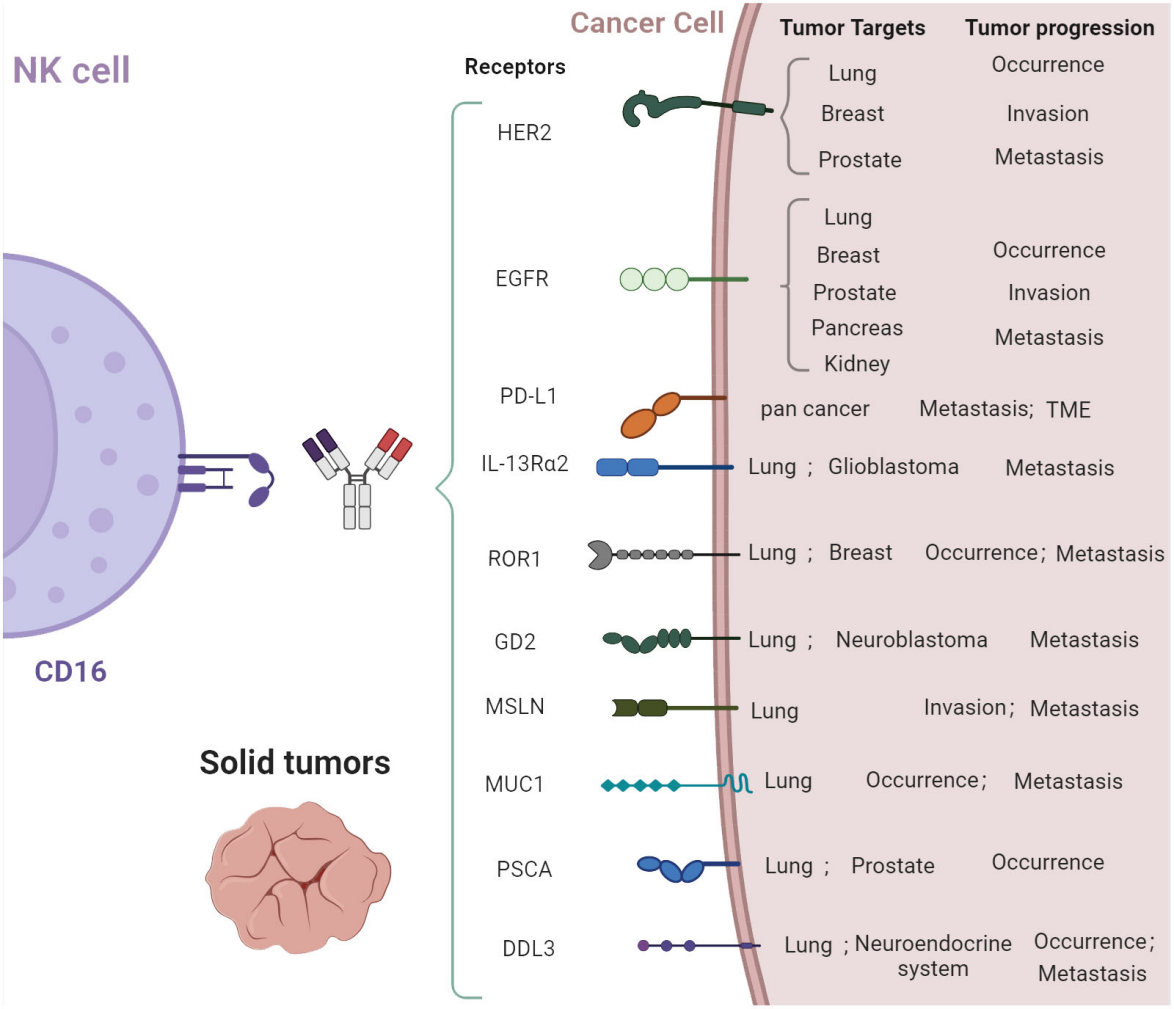
Potential lung cancer targets.

**Figure 2** shows some lung cancer targets that CAR-NK therapy can potentially target.

We have compiled published lung cancer-related targets in CAR-NK engineering therapies and corresponding preclinical research results:

HER2 (human epidermal growth factor receptor 2): HER2 is a well-known target in various cancers, including breast and ovarian cancers. It has also been targeted in lung cancer. Trastuzumab deruxtecan (a HER2 antibody–drug conjugate) showed durable anticancer activity in patients with previously treated HER2-mutant NSCLC (14).
EGFR (epidermal growth factor receptor): EGFR plays a significant role in the development and progression of non-small cell lung carcinoma and other types of cancer. Approximately 15% of NSCLCs express EGFR. CAR T-cell therapies targeting EGFR are being explored and could be adapted for CAR-NK cell therapies in lung cancer (13).
Programmed death ligand 1 (PD-L1) is one of the most successful targets since targeted cancer therapy became popular. Immunotherapies targeted PD-L1, and its receptor (PD-1) have improved survival in a subset of patients with advanced lung cancer (15). Membrane-bound programmed death ligand 1 (PD-L1) level was elevated on a tumor cell surface, which serves as an attractive target for natural killer (NK) cell-mediated therapy (16).
IL-13Rα2 (Interleukin-13 receptor alpha 2): This receptor is highly expressed in glioblastoma but has potential as a target in lung cancer due to its limited expression in normal tissues. It is a negative prognostic factor in human lung cancer and stimulates lung cancer growth in mice (17). IL-13Rα2-targeted therapies have been studied in glioblastoma and could be a novel avenue for lung cancer treatment.
ROR1 (receptor tyrosine kinase-like orphan receptor 1): ROR1 is expressed in many lymphatic and epithelial malignancies and involved in tumor cell survival. It is being examined as a target for CAR T-cell therapies in lung and breast cancers, suggesting potential for CAR-NK cell therapy (18).
GD2 (disialoganglioside GD2): GD2 is highly expressed in neuroblastoma and melanoma but could be considered for SCLC treatment due to its expression on certain small lung cancer cell lines (19). GD2 is one of the few SCLC-specific therapeutic targets. The anti-GD2 antibody dinutuximab was used to treat patients with relapsed/refractory SCLC (20).
7.Mesothelin (MSLN) overexpression is a marker of tumor aggressiveness and associated with reduced recurrence-free and overall survival in early-stage lung cancer (21). Combined with chemokine receptor CCR2b, CAR T treatment targeting MSLN showed good results in the NSCLC mouse model (22).
Mucin 1 (MUC1) is an emerging therapeutic target for solid tumors in recent years. Artificial MUC1-positive animal tumor models show that CAR T therapy targeting MUC1 has a good effect on reducing tumor size and metastasis (23). As shown in a Phase I clinical trial, PD-1 disrupting anti-MUC1-CAR cells achieved a greater response rate and acceptable tolerability results in the NSCLC patients (24).
Prostate stem cell antigen (PSCA) is associated with prostate cancer metastasis. Using aCGH and protein expression analysis, researchers found that PSCA may play an important role in the development of NSCLC brain metastasis and it can be a good therapeutic target for advanced lung tumors (25). Similar with MUC1, PSCA, as a target, performed well in CAR T therapy for NSCLC (26).
Delta-like ligand 3 (DLL3) is an inhibitory Notch ligand that is highly expressed in SCLC and other neuroendocrine tumors but minimally expressed in normal tissues. It is therefore being explored as a potential therapeutic target in SCLC (27).

Additionally, new targets, such as erythropoietin-producing hepatocellular carcinoma A2 (EphA2) (28), tissue factor (TF) (29), and protein tyrosine kinase 7 (PTK7) (30) are currently under clinical investigation. The development of next-generation personalized CAR T cells against solid tumors like NSCLC remains a critical frontier in cancer immunotherapy, given the unique challenges posed by the strong immunosuppressive tumor microenvironment in solid tumors.

The principle of selecting cell therapy targets is the target’s high expression on lung cancer cells and the lack of expression on normal cells to minimize off-target effects. The expression of cell-surface molecules changes in malignantly transformed cells, and CAR-NK cells can target these altered expressions.

## Advantages of CAR NK therapy and other immunotherapy approaches in lung cancer

Immunotherapy has revolutionized cancer treatment by harnessing the body’s immune system to target and destroy cancer cells. Among various immunotherapy modalities, CAR-NK therapy and other approaches offer distinct advantages, paving the way for novel and effective cancer treatments. One of the most significant advantages of CAR-NK therapy over other forms of immunotherapy, such as CAR T-cell therapy, is its inherent ability to target cancer cells with high specificity while minimizing off-target effects. **Table 1** shows comparison of CAR-T and CAR-NK technology. NK cells have a natural ability to distinguish between healthy and malignant cells, reducing the risk of autoimmune responses and collateral damage (31). In particular, CAR-NK therapy has demonstrated promising efficacy in treating solid tumors, including lung cancer, which has been a significant challenge for other forms of immunotherapy. The natural ability of NK cells to infiltrate solid tumors, combined with the specificity of CAR targeting, offers a potent therapeutic approach for lung cancer patients (32).

**Table 1:** The comparison of CAR-NK and CAR T technology.

	CAR T	CAR-NK
Intracellular signaling domain	CD3 ζ with a co-stimulus domain, CD28, etc.	Similar to CAR T structure, but can use NK-specific signal domains such as 2B4, DAP10
Cell source	Autologous or MHC-matched allogeneities	Autologous/allogeneic/NK92 cell line
*In vitro* amplification	Yes	Yes, for autologous NK cells, the cells can be pre-amplified before transduction
Mechanism of killing target cells	CAR-dependent cell killing	CAR-dependent and -independent NK cell-mediated cell killing
Effective time	Effect after antigen presentation	Faster activation does not require antigen presentation
Cytodynamics	Continuous antigen stimulation and increased expression of inhibitory receptors lead to T-cell exhaustion	There is also a problem of NK cell exhaustion
Cytokine release syndrome	Common, usually severe	Not common and not severe
Neurotoxicity	Common	Not common
Invasive tumor characteristics	Usually poor	Usually poor
Developing the potential of ready-to-use products	Limited source of donor cells and the off-the-shelf CAR T cells are being developed	There is potential, but it is necessary to address the efficiency issue of cryopreservation and recovery
Clinical trials	Both the FDA and CFDA have approved a large number of trials	Limited, currently FDA-only approved tumor therapies

Unlike CAR T therapy, CAR-NK therapy demonstrated superior treatment safety. CAR T-cell therapies have been associated with cytokine release syndrome (CRS), a potentially fatal immune response. CAR-NK cells have shown a lower propensity to induce CRS, making CAR-NK therapy a safer alternative (33). This reduced risk enhances patient safety and could lead to broader applicability in clinical settings.

The source of cells in cell therapy often becomes a constraint limiting the clinical application of the treatment. CAR T cells are typically autologous (derived from the patient), which limits its promotion. Currently, many attempts to manufacture off-the-shelf CAR T cells, such as using induced pluripotent stem cells (iPSCs) differentiate T cells (8, 34, 35), are being developed. Hopefully, the source and practicality of CAR T cells can be solved. Unlike T cells, NK cells can be derived from allogeneic sources (donors), making them readily available for “off-the-shelf” use (10, 31). This allogeneic capability significantly reduces the time and cost associated with CAR–NK cell therapy, making it more accessible to a broader patient population. The main sources of NK cells include peripheral blood, umbilical cord blood, NK cell lines, and induced pluripotent stem cells (iPSCs). Peripheral blood is the most traditional source of NK cells for therapeutic purposes. NK cells derived from peripheral blood are readily available and can be collected from the patient (autologous) or a donor (allogeneic). The peripheral blood collection is relatively straightforward, and autologous use minimizes the risk of immune rejection (36). Beyond peripheral blood, umbilical cord blood (UCB) is an alternative source of NK cells that has been explored due to its unique properties. UCB-derived NK cells display a higher degree of immaturity, which might result in better expansion and longevity after infusion. They also carry a lower risk of causing graft-versus-host disease (GvHD) when used allogeneically (37).

In addition to the abovementioned naturally derived NK cells, NK cells obtained through cell-engineering methods have also been proven to be able to load CAR structures to build CAR-NK therapy. The iPSCs represent a cutting-edge source of NK cells, capable of differentiating into any cell type, including NK cells. The iPSCs provide an inexhaustible source of NK cells that can be genetically engineered to enhance their anticancer properties. While the differentiation process is complex and costly, the long-term safety of iPSC-derived NK cells remains to be fully established (38). What is more, several human NK cell lines, such as NK-92, have been genetically modified to express CARs for therapeutic applications^–10^. NK cell lines offer a consistent and unlimited source of NK cells that can be readily engineered and expanded *in vitro*. The use of cell line sources makes the expansion and packaging process easy. At the same time, it can also reduce the batch effect and instability of engineered cells. The use of NK cell lines poses a risk of tumorigenicity, and regulatory authorities require irradiation before infusion, which can affect the longevity and potency of these cells *in vivo* (39).

## Trials, challenges, and future directions of CAR-NK treatment of lung cancer

As of January 2024, researchers have tried many different types of CAR-NK therapies for lung cancer, including two approved clinical trials. A chimeric co-stimulatory transition receptor, (CCCR) consisting of PD1 extracellular domain, NKG2D transmembrane and cytoplasmic domain, and NKG2D cytoplasmic domain 4–1BB, was applied to CAR-NK cell engineering. This receptor can convert negative PD1 signals into activation signals, effectively reversing the immunosuppressive effect of PD1. In a lung cancer xenograft model, CCCR-NK92 cells demonstrated significant inhibition of tumor growth (40). NCT03656705 has been conducted in the First Affiliated Hospital of Xinxiang Medical University, which uses the CCCR-NK92 cells to treat NSCLC patients from 18 to 75 years. For SCLC, Liu et al. engineered DLL3-specific NK-92 cells and explored their potential in treating SCLC. DLL3-CAR NK-92 cells induce tumor regression in an H446-derived lung metastatic tumor model at a favorable safety threshold (41). The DLL3-CAR approach also started a clinical trial in Tianjin Medical University Cancer Institute and Hospital to treat SCLC patients from 18 to 75 years old (NCT05507593). In addition to the abovementioned two studies that have carried out clinical trials, NK-92MI cells carrying an anti-B7-H3 CAR (second-generation CAR) effectively restricted the growth of transplanted non-small cell lung cancer in mice, significantly prolonging the survival time compared to unmodified NK-92MI cells (42). Mesenchymal–epithelial transition factor (C-Met) has been acknowledged as a significant therapeutic target for treating lung adenocarcinoma (LUAD). Anti-c-Met scFv, transmembrane domain of NKG2D, and cytoplasmic signaling domain of CD137, 2B4, DAP10, and CD3ζ were employed to construct four c-Met-CAR structures with different combinations (CC1, CC2, CC3, CC4). The tumor-inhibitory role of c-Met-CAR-NK cells was finally evaluated *in vitro* and *in vivo (* 43).

While these therapies offer promising avenues for improving patient outcomes, they also face significant challenges that must be addressed to fully realize their potential. One of the primary challenges in CAR-NK cell therapy is the immunosuppressive nature of the tumor microenvironment (TME) in lung cancer. The TME can inhibit NK cell activation and function through various mechanisms, including the expression of inhibitory molecules [e.g., PD-L1 (44)] and the presence of immunosuppressive cells [e.g., Tregs (45), myeloid-derived suppressor cells (46)]. Overcoming the immunosuppressive barriers of the TME is crucial for enhancing the efficacy of CAR-NK cell therapies. Besides TME, the persistence and effective trafficking of CAR-NK cells to tumor sites are crucial for their antitumor activity. However, current CAR-NK cell therapies often face challenges with limited *in vivo* persistence and inefficient migration to the tumor site, which can significantly reduce their therapeutic efficacy (32). Tumor heterogeneity and the emergence of antigen escape variants pose significant challenges to CAR-related cell therapies. Similar to the dilemma faced by CAR T therapy, the loss or downregulation of target antigens on tumor cells can lead to the evasion of CAR-NK cell-mediated recognition and destruction, contributing to relapse and treatment resistance (47).

To overcome these difficulties, future CAR-NK cell therapies for lung cancer should be carried out in these following directions: 1. Overcoming TME suppression: future strategies could focus on combining CAR-NK cell therapy with agents that modulate the TME, such as checkpoint inhibitors [e.g., anti-PD-1, anti-PD-L1 antibodies (48)] or agents targeting the suppressive cells within the TME. These combinations could enhance the efficacy of CAR-NK cells by reducing immunosuppression and promoting a more favorable tumor microenvironment for NK cell activity. 2. Enhancing CAR NK cell persistence and trafficking: advancements in genetic engineering could lead to the development of CAR-NK cells with enhanced persistence and trafficking capabilities. For example, incorporating genes that encode for chemokine receptors matching the chemokines secreted by tumor cells can improve the homing of CAR-NK cells to the tumor site. Additionally, genetic modifications that promote the survival and proliferation of NK cells *in vivo* could improve their persistence and antitumor activity. 3. Targeting multiple antigens: to counteract antigen escape, developing CAR-NK cells that target multiple tumor-associated antigens could be a promising approach. Designing multi-target CAR structures (third-generation CAR structure) could reduce the likelihood of tumor cells evading CAR-NK cell-mediated detection and destruction potentially leading to more durable responses (32, 40, 48). 4. Advancements in manufacturing technologies: innovations in bioprocessing and genetic engineering could streamline the production of CAR-NK cells, making it more efficient and cost effective. The development of “off-the-shelf” CAR-NK cell products from universal donor cells or induced pluripotent stem cells (iPSCs) could also address scalability issues and improve accessibility to CAR-NK cell therapies (38).

## Conclusion

Lung cancer’s high mortality rate and global prevalence underscore the importance of continued research and development in its detection, treatment, and prevention. Primary prevention, like smoking cessation and reducing exposure to environmental risk factors, remains crucial. Advances in medical treatments and early detection strategies offer hope for improved outcomes in lung cancer patients. CAR- NK cell therapy represents a promising avenue for the treatment of lung cancer offering potential advantages over traditional therapies, including reduced risk of CRS and GvHD, and the ability to target and kill cancer cells through innate immune mechanisms. However, addressing the challenges of TME suppression, CAR-NK cell persistence, antigen escape, and scalability is essential for the successful development and clinical implementation of these therapies. Future research and technological advancements will play a critical role in overcoming these obstacles and unlocking the full therapeutic potential of CAR-NK cell therapy for patients with lung cancer.

This review highlights the importance of continued research and collaboration across disciplines to address the complex challenges facing CAR-NK cell therapy in lung cancer. By leveraging advances in genetic engineering, immunology, and manufacturing technologies, there is significant potential to improve the efficacy, safety, and accessibility of CAR-NK cell therapies, offering new hope to patients with lung cancer.

## Author contributions

XS: Investigation, Supervision, Writing – original draft, Writing – review & editing. FL: Writing – original draft, Writing – review & editing. XM: Data curation, Methodology, Writing – review & editing. LH: Investigation, Methodology, Writing – review & editing.
